# Editorial: Neuroinflammation and Cognition

**DOI:** 10.3389/fnagi.2018.00413

**Published:** 2018-12-11

**Authors:** Ashok Kumar

**Affiliations:** Department of Neuroscience, McKnight Brain Institute, University of Florida, Gainesville, FL, United States

**Keywords:** aging, inflammation, oxidative stress, cytokines, cognitive decline

Aging is a complex process, and one of the major risk factors for onset and progression of various neurodegenerative diseases. Increased human longevity has magnified the negative impact that aging can have on cognitive performance. The number of individuals 65 years or older will increase exponentially over the next several decades, and age-related cognitive impairment is expected to have a detrimental impact on individuals, their families, and society. An early sign of age-related cognitive decline in humans and rodent models is impaired executive function and spatial memory performance. Synaptic plasticity in the mammalian central nervous system has been the subject of intense investigation, and is regarded as a principal candidate for cellular mechanisms involved in learning and memory. Senescent physiology including altered synaptic plasticity and cell excitability contributes to the decline in cognitive function associated with aging and age associated neurodegenerative diseases. Neuroinflammation is a common feature of virtually every central nervous system disease, and is being increasingly recognized as a potential mediator of cognitive impairments. Systemic inflammation levels are increased with advanced age and neurodegeneration. The impact of age on neuroinflammatory responses including glial activation, increased production of proinflammatory cytokines, and aberrant neuronal signaling could magnify the deterioration of the central nervous system microenvironment in disease, and may contribute to accelerated cognitive impairment. However, a clear mechanistic understanding of these interactions is lacking. Given the high complexity of aging process and various mechanisms contributing to age-associated cognitive impairment, it is an arduous task to pinpoint on any single factor. This research topic in the *Frontiers in Aging Neuroscience* has produced a highly informative collection of original research articles (13), reviews (two), and a general commentary that cover comprehensive aspect of neuroinflammation and possible therapeutic interventions in rescuing cognitive impairments.

First, in their original research article, Ianov et al. provided detailed research findings related to gene expression profile in hippocampal sub regions and their association with aging and cognition.The authors employed two next-generation RNA sequencing platforms, Illumina, and Ion Proton, to examine gene expression differences related to brain aging and cognitive impairment in CA1, CA3, and dentate gyrus of the hippocampus. Their results demonstrate differences in gene expression in hippocampal sub regions and indicate regional differences in susceptibility to advanced age and cognitive performance. Rani et al. reported their exciting results in regards to miRNA in circulating microvesicles as being biomarkers for age-related cognitive deficit. The authors demonstrated that miRNA, from extracellular microvesicle enriched plasma samples, correlates with cognitive status in healthy elderly individuals. Bradburn et al. published their results in regards to elevated biomarkers of systemic inflammation and cognitive decline. The authors performed meta-analysis of prospective studies investigating the relationship between established markers of peripheral inflammation, the interleukin-6, with risk of cognitive impairment. Their results provide an evidence that individuals with high baseline interleukin-6 are more likely to develop cognitive decline. Rai et al. investigated the protective role of aqueous extract of Mucuna pruriens (Mp) against 1-methyl-4-phenyl-1,2,3,6-tetrahydropyridine (MPTP)-induced neuroinflammation and behavioral abnormalities, and delineated possible signaling pathways. The authors found that oral administration of MPTP in mice significantly and negatively influenced the behavioral performance and induced an increase in various inflammatory markers including glial fibrillary acidic protein, intercellular cell adhesion molecule, and tumor necrosis factor alpha in substantia nigra pars compacta. Treatment with Mp significantly reduced these inflammatory markers. The authors conclude that administration of Mp extract reversed MPTP-induced neuroinflammation and behavioral abnormalities by activating nuclear factor kB/pAKT signaling pathways. Nusrat et al. illustrated with their novel research on the role of acute and chronic cyclosporine A treatment on activation of endogenous neural precursor cells, neuroprotection, and tissue damage. The authors demonstrated that long-term treatment with cyclosporine A activated neural precursor cells, stimulated migration of neural precursor cells to the site of injury, and ameliorated cognitive recovery. Lee et al. in their original research investigated beneficial effects of Antler combined with Liuwei Dihuang pills (ALWPs) on cognition and lipopolysaccharide (LPS)-induced neuroinflammation. The authors found that oral administration of ALWPs rescued LPS-induced short and long-term memory impairment and attenuated microglial activation, possibly by modulating toll-like receptor/focal adhesion kinase/nuclear factor-kB signaling pathways. Hei et al. investigated the expression profile of growth factor neuregulin 1 (NRG1) and tyrosine kinase receptor ErbB4 in the CA1 region of the hippocampus following chronic cerebral hypoperfusion, and their possible relationship with neuronal degeneration and glial activation. The authors demonstrated that the expression of NRG1 and ErbB4/phospho ErbB4 peaked during acute phase and then decreased in the chronic phases of cerebral hypoperfusion. The expression of NRG1/ErbB4 in the CA1 region of the hippocampus was positively correlated with the degree of neuronal apoptosis, but not with glial activation. The study by Yang et al. investigated the influence of naringin dihydrochalcone on cognitive performance and the neuropathology of an Alzheimer's mouse model. Their results illustrate that oral administration of naringin dihydrochalcone ameliorated cognitive function, reduced amyloid plaque burden/Aβ levels, attenuated neuroinflammation, and augmented neurogenesis. The paper by Lin et al. provides evidence for a mediating role of systemic inflammation on the association between age and cognitive function. The study assessed serum concentrations of three inflammatory biomarkers, interleukin 6 (IL-6), tumor necrosis factor alpha, and C-reactive protein, as well as measured processing speed and short-term memory via performance-based tests in 47 young and 46 older generally healthy adults. A mediation analysis showed that the level of IL-6 partially accounted for differences in processing speed between young and older participants. IL-6 also mediated age-related impairment in processing speed within the older but not in the young participant group. The study observed no associations between any of the inflammatory biomarkers and short-term memory. Klein et al. in their innovative research investigated whether microglia in the middle-aged central nervous system responded differently to demyelination process. The authors reveal that in middle-aged animals, microglia, the resident immune cells of the central nervous system, are already altered, and react differently to demyelination. Cichoń et al. in their work evaluated the influence of extremely low-frequency electromagnetic field therapy on neuroplasticity in the rehabilitation of patients following moderate stroke. The authors report that electromagnetic field therapy significantly improved functional recovery in post-stroke patients by ameliorating neuroplasticity processes including upregulation of neurotrophic factors and plasma cytokines. In their original research article, Tiwari et al. explored for the first time the neuroprotective influence of withaferin A against amyloid beta pathology. The authors observed that amyloid beta significantly damaged the neuronal function and morphology, and withaferin A reduced the amyloid beta-induced neurotoxicity.

Rai et al. published their commentary on an article entitled “mild endoplasmic reticulum stress ameliorates LPS-induced neuroinflammation and cognitive impairment via regulation of microglial polarization” by Wang et al. (2017), suggesting that mild to moderate level of endoplasmic stress could provide an alternative therapeutic to delay neuroinflammatory-induced neurodegenerative diseases (Rai et al.).

In their review article, Musella et al. provided an overview of clinical and experimental studies emphasizing the various impacts of advanced age on motor disability and cognitive impairment in multiple sclerosis. The authors raised challenging questions on the putative age-related mechanisms including neuroinflammatory processes in contributing to the onset and progression of neurodegenerative processes related to multiple sclerosis. Sompol and Norris in their well-designed review article described age-associated alterations in Ca^2+^ regulation and the relation of these alterations to neurodegenerative diseases. The authors described that changes in Ca^2+^ regulation are usually attributed to neurons and are commonly discussed in the context of neuronal signaling pathways. However, astrocytes also exhibit striking aging and disease-related changes in Ca^2+^ regulation, especially in regions of marked pathology. The authors further report that the altered expression and activity of the Ca^2+^-dependent protein phosphatase, calcineurin, in activated astrocytes is a function of age, injury, and disease. Calcineurin is found in a proteolized and highly activated state in astrocytes associated with amyloid deposits and damaged or occluded cerebral blood vessels. Blockade of the calcineurin-dependent transcription factor, the nuclear factor of activated T cells-selectively activated astrocytes, improved synaptic function, reduced excitotoxic damage, and stabilized cognition in a variety of rodent models of injury and disease. Finally, the authors conclude that Ca^2+^ dysregulation in activated astrocytes results in neurologic dysfunction due to hyper activation of calcineurin.

Aging is characterized by a progressive increase in neuroinflammation, which contributes to cognitive impairment, associated with aging and age-related neurodegenerative diseases including Alzheimer's. However, mechanisms linking neuroinflammation and cognitive impairment are not yet clearly elucidated. Overall, this research topic attracted plenty of interesting original research articles as well as few reviews, and a general commentary, which brought comprehensive knowledge of age-related neuroinflammation and cognitive decline. This collection of remarkable articles delivered detail information on age-associated enhanced neuroinflammation and its influence on cognition. Finally, I hope that this research topic has provided understandings of therapeutic approaches for the treatment of aging-associated neuroinflammation-induced cognitive impairment.

## Author Contributions

The author confirms being the sole contributor of this work and has approved it for publication.

### Conflict of Interest Statement

The author declares that the research was conducted in the absence of any commercial or financial relationships that could be construed as a potential conflict of interest.
